# Real-World Experience of Pembrolizumab Monotherapy in Patients with Recurrent or Persistent Cervical Cancer: A Korean Multi-Center Retrospective Study (KGOG1041)

**DOI:** 10.3390/cancers12113188

**Published:** 2020-10-29

**Authors:** Min Chul Choi, Yong-Man Kim, Jeong-Won Lee, Yong Jae Lee, Dong Hoon Suh, Sung Jong Lee, Taek Sang Lee, Maria Lee, Dong Choon Park, Min Kyu Kim, Jong-Min Lee, Seung-Hyuk Shim, Seob Jeon, Kyung Jin Min, Mi Kyung Kim, Bo Wook Kim, Jeong Yeol Park, Byoung-Gie Kim, Dae Yeon Kim, Moon-Hong Kim, Hyun-Soo Kim, Jung-Yun Lee

**Affiliations:** 1 Comprehensive Gynecologic Cancer Center, CHA Bundang Medical Center, CHA University, Seongnam-si, Gyeonggi-do 13496, Korea; 2 Department of Obstetrics and Gynecology, University of Ulsan College of Medicine, Asan Medical Center, Seoul 05505, Korea; ymkim@amc.seoul.kr (Y.-M.K.); obgyjypark@amc.seoul.kr (J.Y.P.); kdyog@amc.seoul.kr (D.Y.K.); 3 Department of Obstetrics and Gynecology, Samsung Medical Center, Sungkyunkwan University School of Medicine, Seoul 06351, Korea; bgkim@skku.edu; 4 Department of Obstetrics and Gynecology, Yonsei University College of Medicine, Seoul 03722, Korea; SVASS@yuhs.ac (Y.J.L.); jungyunlee@yuhs.ac (J.-Y.L.); 5 Department of Obstetrics and Gynecology, Seoul National University Bundang Hospital, Seongnam 13620, Korea; sdhwcj@snubh.org; 6 Department of Obstetrics and Gynecology, Seoul St. Mary’s Hospital, College of Medicine, The Catholic University of Korea, Seoul 06591, Korea; orlando@catholic.ac.kr; 7 Department of Obstetrics and Gynecology, SMG-SNU Boramae Medical Center, Seoul 07061, Korea; tslee70@snu.ac.kr; 8 Department of Obstetrics and Gynecology, Seoul National University College of Medicine, Seoul 03080, Korea; marialee@snu.ac.kr; 9 Department of Obstetrics and Gynecology, St. Vincent’s Hospital, The Catholic University of Korea, Suwon 16247, Korea; dcpark@catholic.ac.kr; 10 Division of Gynecologic Oncology, Department of Obstetrics and Gynecology, Samsung Changwon Hospital, Sungkyunkwan University School of Medicine, Changwon 51353, Korea; minkyukim@skku.edu; 11 Department of Obstetrics and Gynecology, Kyung Hee University Hospital at Gangdong, Seoul 05278, Korea; jmleemd@khu.ac.kr; 12 Department of Obstetrics and Gynecology, Research Institute of Medical Science, Konkuk University School of Medicine, Seoul 05030, Korea; 20130131@kuh.ac.kr; 13 Department of Obstetrics and Gynecology, Soonchunhyang University College of Medicine, Cheonan 31151, Korea; sjeon@schmc.ac.kr; 14 Department of Obstetrics and Gynecology, Korea University Medical Center, Korea University College of Medicine, Seoul 15355, Korea; mikji97@korea.ac.kr; 15 Department of Obstetrics and Gynecology, Ewha Womans University College of Medicine, Seoul 07985, Korea; asterik79@ewha.ac.kr; 16 Department of Obstetrics and Gynecology, International St. Mary’s Hospital, Catholic Kwandong University College of Medicine, Incheon 22711, Korea; kimbw@ish.ac.kr; 17 Department of Obstetrics and Gynecology, Korea Cancer Center Hospital, Korea Institute of Radiological & Medical Sciences, Seoul 01812, Korea; garymh@kcch.re.kr; 18 Department of Pathology and Translational Genomics, Samsung Medical Center, Sungkyunkwan University School of Medicine, Seoul 06351, Korea; hyun-soo.kim@samsung.com

**Keywords:** cervical cancer, immune checkpoint inhibitor, pembrolizumab, recurrence

## Abstract

**Simple Summary:**

Immune checkpoint inhibitors have received considerable interest because of their ability to generate durable response in many intractable malignant solid tumors. The therapeutic results of immune checkpoint inhibitors in recurrent or advanced uterine cervical cancer, which associated with persistent infection with human papillomavirus, from several well-designed clinical trials are reported. However real-world experiences are not yet provided. In this study, we retrospectively assessed the efficacy and safety of pembrolizumab, one of the immune checkpoint inhibitors, in real-world practice among patients in Korea with recurrent or persistent cervical cancers. The results of this study show modest antitumor activity comparable to that found in previously reported clinical trials. Although in patients with favorable performance status, pembrolizumab showed effective antitumor activity. Some safety profiles should be carefully monitored during treatment.

**Abstract:**

This study investigated the antitumor activity and safety of pembrolizumab in patients with recurrent cervical cancer in real-world practice. We conducted a multi-center retrospective study of patients with recurrent or persistent cervical cancer treated with pembrolizumab at sixteen institutions in Korea between January 2016 and March 2020. The primary endpoints were the objective response rate (ORR) and safety. Data were available for 117 patients. The median age was 53 years (range, 28–79). Sixty-four (54.7%) patients had an Eastern Cooperative Oncology Group (ECOG) performance status of ≥2. Forty-nine (41.9%) patients were stage ≥III at diagnosis. Eighty-eight (75.2%) patients had squamous cell carcinoma. The median number of prior chemotherapy lines was two (range, 1–6). During the median follow-up of 4.9 months (range, 0.2–35.3), the ORR was 9.4%, with three complete responses and eight partial responses. The median time to response was 2.8 months (range 1.3–13.1), and the median duration of response (DOR) was not reached. In the population of patients with favorable performance status (ECOG ≤1) (*n* = 53), the ORR was 18.9%, and the median DOR was 8.9 months (range, 7.3–10.4). Adverse events occurred in 55 (47.0%) patients, including eight (6.8%) patients who experienced grade ≥3 events, and two of them were suspicious treatment-related deaths. Pembrolizumab had modest antitumor activity in patients with recurrent cervical cancer comparable to that found in previously reported clinical trials. However, in patients with favorable performance status, pembrolizumab showed effective antitumor activity. Some safety profiles should be carefully monitored during treatment.

## 1. Introduction

Uterine cervical cancer is one of the most common gynecologic cancers and the fourth leading cause of cancer-related death in women worldwide. More than 569,847 women were diagnosed with cervical cancer in 2018, resulting in more than 311,365 deaths [1]. It is estimated that 3148 new cases of cervical cancer and 801 cancer-related deaths will occur in Korea in 2020 [2]. For recurrent or metastatic cervical cancer, bevacizumab in combination with cisplatin-based chemotherapy is currently the standard treatment, and this approach provides a median survival of approximately 17 months [3]. Therapeutic options are limited for patients who progress after initial therapy to recurrent or metastatic cervical cancer.

It has been established that persistent infection with human papillomavirus (HPV) is associated with the carcinogenesis of cervical cancer [4] and HPV infections account for as much as 86% of the worldwide incidence of cervical cancer [5]. Two viral oncogenes, E6 and E7, play a major role in the malignant transformation of HPV-infected cervical cells. These viral antigens are consistently expressed in HPV-induced neoplasm and might represent attractive targets for cancer immunotherapy [6,7]. Cancer cells can induce immune evasion by immune checkpoints such as cytotoxic T-lymphocyte antigen 4 or programmed death-1 (PD-1), allowing them to escape from the tumor-specific T-cell response [8]. These negative signals in several solid tumors have been shown to be provided mainly by PD-1, and programmed death-ligand 1 (PD-L1) expression was recently identified in more than half of cervical cancers [9,10]. Therefore, using a monoclonal antibody to inhibit PD-1/PD-L1 co-inhibitory pathways might be an effective therapeutic approach to reverse immune suppression and to activate a cancer-specific immune response in cervical cancer patients. 

Several clinical trials have been conducted to explore the application of PD-1 inhibitors to cervical cancer. The objective response rate to these PD-1 inhibitors (i.e., pembrolizumab and nivolumab) in patients with advanced or recurrent cervical cancer was reported to be 4%–26% [11,12,13,14]. Based on these results, the U.S. Food and Drug Administration approved pembrolizumab in June 2018 for the treatment of patients with PD-L1-positive recurrent or metastatic cervical cancer. However, real-world efficacy data are limited.

In Korea, pembrolizumab can be offered off-label to patients with recurrent squamous cell cervical cancer and PD-L1-positive recurrent cervical cancer. In this study, we retrospectively assessed the efficacy and safety of pembrolizumab in real-world practice among patients in Korea with recurrent or persistent cervical cancers.

## 2. Materials and Methods

### 2.1. Study Design and Patients 

We conducted a multi-center, retrospective study at sixteen institutions affiliated with the Korean Gynecologic Oncology Group (KGOG). We reviewed the clinicopathologic and radiologic records of women diagnosed with recurrent or persistent uterine cervical cancer who were treated with pembrolizumab between January 2016 and March 2020. The inclusion criteria were as follows: (1) histologically confirmed cervical cancer; (2) tumor progression during or after the use of one or more lines of chemotherapy with measurable disease, irrespective of Eastern Cooperative Oncology Group (ECOG) performance status; (3) use of pembrolizumab for at least one treatment cycle. Patients were administered 200 mg of pembrolizumab as 30-min intravenous infusions every 3 weeks until disease progression, unacceptable toxicity, or patient withdrawal occurred. This study was approved by the Institutional Review Board at each participating institution (CHA IRB 2019-11-003) and adhered to the principles in the Declaration of Helsinki. 

Pathologic information, including histology and results of PD-L1 staining, was collected, and some institutions additionally performed a PD-L1 test. Tumor PD-L1 expression was analyzed using the PD-L1 IHC 22C3 antibody (Dako, Santa Clara, CA) to determine the tumor proportion score (TPS), defined as the percentage of viable tumor cells, or using the PD-L1 IHC 22C3 pharmDx assay (Agilent Technologies, Carpinteria, CA) to determine the combined positive score (CPS), defined as the ratio of PD-L1-positive cells (tumor cells, lymphocytes, and macrophages) to the total number of viable tumor cells multiplied by 100. PD-L1 positivity was defined as a TPS ≥1% or a CPS >1.

The datasets supporting the conclusions of this article are included within the article and its additional images. Raw data are available from the corresponding author upon reasonable request.

### 2.2. Assessments

Tumor imaging was basically performed by abdomino-pelvic and/or chest computed tomography (CT) every 9 weeks. If clinical symptoms were deteriorated, image studies were performed immediately at the clinician’s discretion. Pelvic magnetic resonance imaging, whole body bone scan, or positron emission tomography/CT scans were performed when indicated. The tumor response assessment was performed according to the Response Evaluation Criteria in Solid Tumors (RECIST) version 1.1 by gynecologic oncologists. Safety was assessed by retrospective chart review of laboratory tests and physical examinations were performed before each treatment cycle to detect any possible adverse events (AEs), which were evaluated according to the Common Terminology Criteria for Adverse Events version 4.03. (http://www.oncology.tv/SymptomManagement/NationalCancerInstituteUpdatesCTCAEtov403.aspx).

### 2.3. Primary and Secondary Objectives

The primary endpoints were the objective response rate (ORR), defined as the proportion of patients with a complete response (CR) or a partial response (PR), as assessed by RECIST version 1.1, and the rate of any AEs. The secondary endpoints were the duration of response, defined as the time from the response to tumor progression or death, whichever occurred first; progression-free survival (PFS), defined as the time from the start of pembrolizumab to tumor progression or death; overall survival (OS), defined as the time from the start of treatment to death from any cause. An additional efficacy analysis was conducted in the subgroup of patients with favorable performance status (ECOG ≤ 1). 

ORR point estimates, accompanied by 95% confidence intervals (CIs), were calculated using the Clopper–Pearson exact method. Patients without response data were considered to be non-responders. Duration of response, PFS, and OS were estimated using the Kaplan–Meier method. To identify factors affecting the ORR, univariate logistic regression analyses were performed. A further multivariate logistic regression analysis was intended to use factors with a significance level of less than 0.1 in the univariate analyses.

## 3. Results

### 3.1. Patients

**Figure 1:**
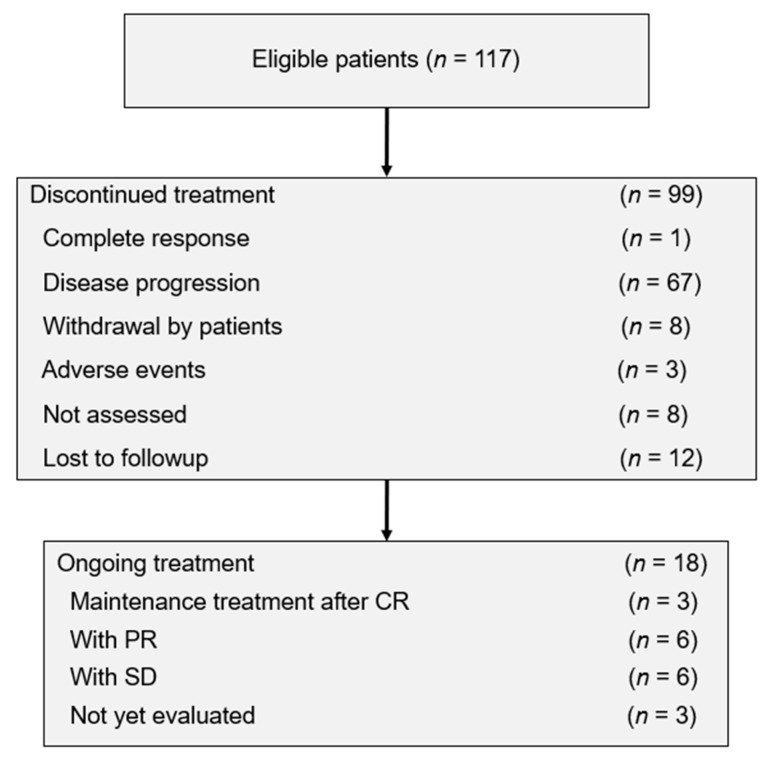
Patient disposition. CR, complete response; PR, partial response; SD, stable disease.

Information for 117 patients treated with pembrolizumab was collected from 16 sites affiliated with KGOG. One patient diagnosed with vaginal cancer and two duplicate patients were excluded (Table S1). The clinicopathologic characteristics of the patients are listed in Table 1. The median age was 53.0 years (range, 28–79 years). Of the patients, 45.3% (53/117) had an ECOG performance status (PS) of ≤1, and 41.9% (49/117) had stage III or IV at diagnosis. The HPV test was done in 71 patients (60.7%), of whom 57 (80.3%) were HPV-positive. PD-L1 expression was tested in 72 patients (61.5%), of whom 60 (83.3%) were PD-L1-positive by TPS or CPS. Eighty-eight (75.2%) patients had squamous cell carcinoma histology. Fifty patients (42.7%) had received previous radiotherapy, and 62 patients (53.0%) had received previous surgery. The median number of prior chemotherapy lines, including neoadjuvant chemotherapy, was two (range, 1–6). As of 31 March, 2020, the data cutoff, the median follow-up time was 4.9 months (range, 0.2–35.3 months). Ninety-nine patients (84.6%) had discontinued pembrolizumab, most commonly due to disease progression (57.3%; *n* = 67) (Figure 1). The median number of pembrolizumab cycles was three (range, 1–24 cycles).

### 3.2. Antitumor Activity

**Figure 2:**
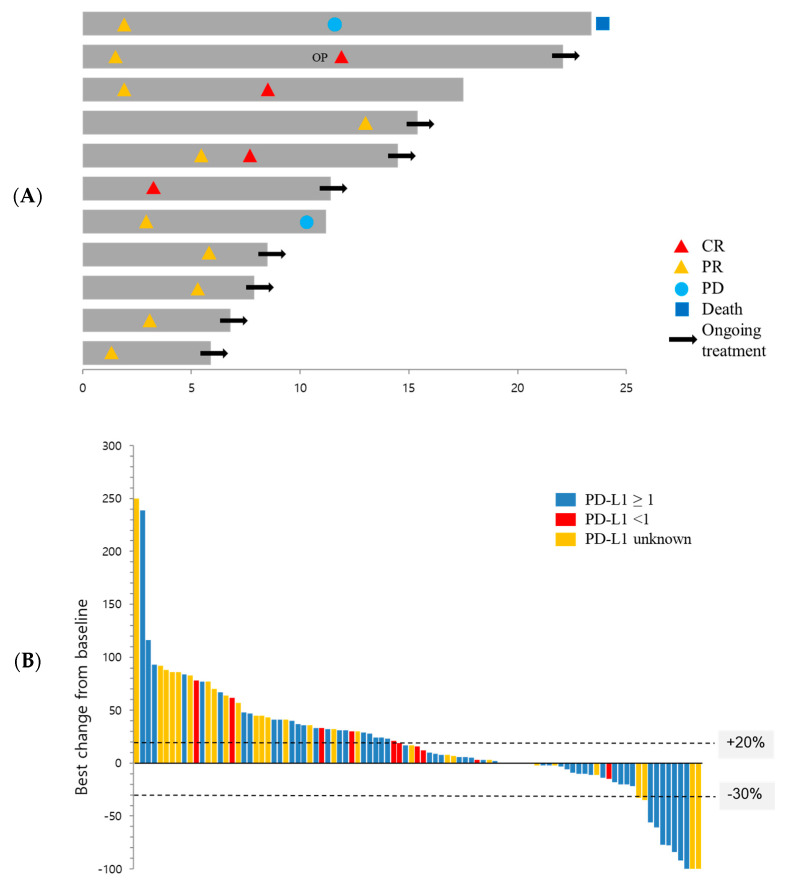
Antitumor activity of pembrolizumab. (**A**) Time to and duration of response in patients whose best overall response was CR or PR (*n* = 11). The length of bars represents the time to the last image assessment. (**B**) Waterfall plot showing the distribution of the best percentage change in the sum of the target lesion size from baseline according to Response Evaluation Criteria in Solid Tumors (RECIST) version 1.1 (*n* = 95). OP, operation; CR, complete response; PR, partial response; PD, progressive disease; PD-L1, programmed death-ligand 1.

In the total population, three patients (2.6%) achieved a CR and eight (6.8%) achieved a PR, resulting in an ORR of 9.4% (95% CI, 4.8–16.2) (Table 2). A clinical summary of the 11 responders is provided in Table S2. Ten of the 11 responders had squamous cell carcinoma (one, BCHA013, had adenosquamous histology) and 10 had favorable ECOG PS (one, BCHA006, had a PS of 3). The median time to response was 2.8 months (range, 1.3–13.1 months) and the median duration of response was not reached (range, 8.9–not reached). Eight of the 11 responders were still receiving pembrolizumab at the data cutoff date (Figure 2A). In the population of patients (*n* = 53) with favorable PS, the ORR was 18.9% (95% CI, 9.4–32.0) (Table 2). The median time to response in that group was 3.0 months (range, 1.3–13.1 months), and their median duration of response was 8.9 months (range, 7.3–10.4 months). 

Twenty-eight patients (23.9%) in the total population and 14 patients (26.4%) in the favorable PS group showed stable disease, leading to disease control rates of 33.3% (95% CI, 24.9–42.6) and 45.3% (95% CI, 31.6–59.6), respectively (Table 2). The best percentage change in target lesion from baseline among the 95 patients with one or more evaluable post-baseline imaging assessments is shown in Figure 2B. 

**Figure 3:**
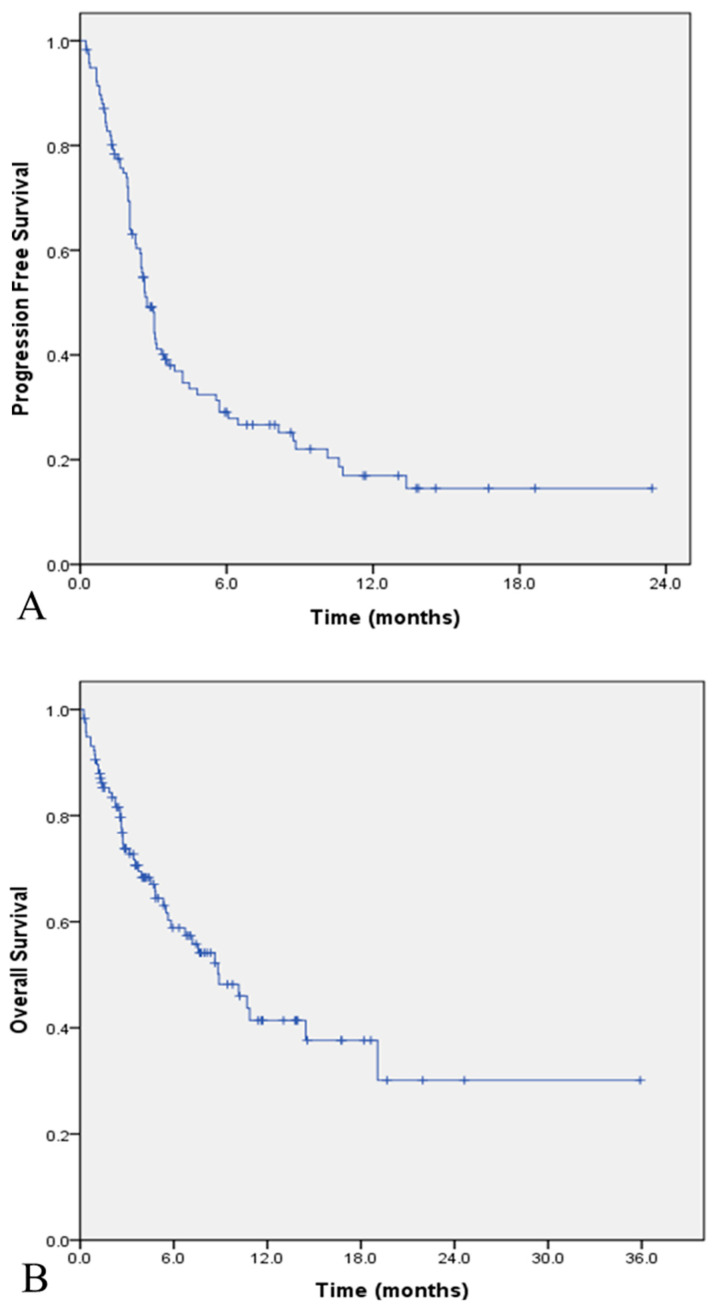
Kaplan–Meier estimates of survival in the total study population (*n* = 117). (**A**) Progression-free survival; (**B**) overall survival.

At the time of data cutoff, 81 (69.2%) patients in the total population had experienced disease progression or death. The median PFS was 2.7 months (95% CI, 2.3–3.1 months), and the estimated PFS rates at six and 12 months were 29.6% and 16.6%, respectively (Figure 3A). In the favorable PS group, which had 32 patients with disease progression (60.4%), the median PFS was 4.5 months (95% CI, 1.8–7.2 months; Figure S1A). A total of 53 patients (45.3%) in the total population and 15 (28.3%) in the favorable PS group had died. The median OS was 8.8 months (95% CI, 5.6–12.1 months) in the total population (Figure 3B) and 19.1 months (95% CI, 2.5–35.6 months) in the favorable PS group (Figure S1B). The six-month estimates of OS were 58.6% and 84.1%, respectively, and the 12-month estimates were 41.1% and 57.3%, respectively. One of the 11 responders (BCHA010) expired due to cancer progression (Table S2).

### 3.3. Safety

A total of 55 patients (47.0%) experienced one or more adverse events, including eight (6.8%) patients who experienced grade ≥3 events (Table 3). Three patients (2.6%) discontinued pembrolizumab due to AEs, including two whose deaths were suspected to have resulted from treatment-related AEs. One of these patients, who suddenly developed pneumonitis with pulmonary edema 20 days after the first cycle, refused further management for the AE, and expired the day after symptom manifestation. The second patient, who had grade 4 colitis after three cycles, also refused further management for the AE, and died 10 days later. The third patient discontinued treatment due to the occurrence of grade 3 Guillain–Barré syndrome; the patient recovered from these symptoms, but received no further pembrolizumab. The most common AEs for any grade were hypothyroidism (7.7%), fatigue (4.3%), and skin rash (4.3%).

### 3.4. Prognostic Factors

Analyses examining how HPV positivity, histology, the number of previous lines of chemotherapy, ECOG status, PD-L1 positivity, and burden of tumor affected ORR were performed using logistic regressions (Table 4). Favorable ECOG PS (≤1) was the only significant factor in the univariate regression analyses (odds ratio, 14.651; 95% CI, 1.809–118.675; *p* = 0.012), so no further multivariate regression analysis was done.

## 4. Discussion

### 4.1. Antitumor Activity

The results of this study show modest antitumor activity for pembrolizumab in patients with recurrent or persistent cervical cancer. The ORR was 9.4%, with three patients achieving a CR and eight patients a PR. The median PFS was 2.7 months, and the estimated PFS rate at six months was 29.6%. The median OS was 8.8 months, and the six- and 12-month estimates for OS were 58.6% and 41.1%, respectively. The therapeutic results from PD-1 inhibitors monotherapy in recurrent or advanced cervical cancer are summarized in Table 5 [11,12,13,14]. Although it is difficult to directly compare our results with those from well-designed clinical trials, they are generally consistent. As in a previous report, when we analyzed the 88 patients with squamous cell carcinoma, which is known to have a relatively favorable prognosis, their ORR was 11.4% (Table S3). The results from prospective clinical trials and our results from this retrospective study differ in some points. The prospective studies could only enroll patients with a favorable PS (ECOG ≤1), but in real-world practice, patients with poor general condition (ECOG ≥2) are also treated. In the real world, immune checkpoint inhibitors (ICIs) tend to be tried as a last attempt for heavily treated patients in poor general condition. In this study, more than half (54.7%, 64/117) of the patients had an ECOG ≥2 (Table 1), which well reflects real-world clinical circumstances.

In this context, the promising antitumor activity of pembrolizumab could be assessed by analyzing the treatment response in only the favorable PS group in this study. The ORR in this group (*n* = 53) was 18.9%, and their disease control rate was 45.3% (Table 2). Furthermore, their median PFS was 4.5 months (95% CI, 1.8–7.2 months), with an estimated PFS rate of 44% at six months. Their median OS was 19.1 months (95% CI, 2.5–35.6 months), and the estimated six- and 12-month estimates of OS were 84.1% and 57.3%, respectively (Table 5). These results indicate that pembrolizumab has antitumor activity that is somewhat better than that reported in the KEYNOTE-158 study [14], which used the same dose of pembrolizumab. Therefore, the results of this real-world study indicate that among patients with recurrent or persistent cervical cancer, pembrolizumab treatment showed better antitumor activity in patients with favorable PS.

In addition, as in the results of the CheckMate 358 trial [12] of nivolumab, patients treated with less than two previous chemotherapy lines had an ORR of 26% (Table 5), suggesting that a low number of prior treatments improved the response. As previously suggested by Tewari [15], treatment with ICIs could be a second-line therapeutic option for patients with progressed cervical cancer who have failed with standard therapy after confirmation of PD-L1 expression. An ongoing phase III randomized trial is investigating chemotherapy with ICIs as a first-line treatment for chemo-naïve patients with persistent, recurrent, or metastatic cervical cancer [16,17]. 

### 4.2. Adverse Events

According to a recent meta-analysis [18], AEs of any grade occurred in 65.8% of patients receiving an ICI, and 16.6% of patients experienced grade ≥3 AEs. Treatment discontinuation due to AEs occurred in 6.4% of patients, and treatment-related death (TRD) was less common in patients treated with ICIs (0.9%) than in patients treated with chemotherapy (1.3%). To date, TRD has not been reported in studies of cervical cancer patients [11,12,13,14]. In the present study, an AE occurred in 47.0% of patients, and 6.8% of patients experienced grade ≥3 AEs. Treatment discontinuation due to AEs occurred in 2.6% of patients, and suspicious TRD occurred in two (1.5%) patients. 

Compared to the reported clinical trials, the frequency of AEs in this study was relatively low, which is likely due to the limitations of a retrospective study conducted using chart reviews, because minor AEs might not have been recorded. In contrast, 1.5% (*n* = 2) of patients were suspected of TRD. These patients might not have died if they had been adequately managed for their AEs. Furthermore, it was difficult to identify the accurate cause of death in these patients because no autopsy or additional investigation was conducted. The most commonly reported causes of TRD are known to be immune-related pneumonitis and intestinal perforation/colitis [18]; therefore, clinicians have to be fully aware of these risks when prescribing ICIs. 

### 4.3. Prognostic Marker

Microsatellite instability-high and/or deficient DNA mismatch repair (MSI-H/MMRd) has been identified as a potential predictive marker for response to ICIs [19,20,21]. Regardless of the type of cancer, solid tumors showing MSI-H/MMRd are known to have a favorable response to pembrolizumab, with an ORR of 34%–53% [19,21]. However, tumors with MSI-H/MMRd represent only 2%–4% of all diagnosed cancers [22,23]. In gynecologic cancers, MSI-H/MMRd tumors are found in 22%–33% of all endometrial cancers, and only 10% of all ovarian and cervical cancers [19,20]. Therefore, other prognostic markers, such as PD-L1 expression, tumor mutational burden, and clinical biomarkers, have been studied. Of them, PD-L1 expression on either tumor or immune cells has emerged as an alternative predictive biomarker. The ORR was higher in patients with PD-L1-positive cervical cancers than in the overall population (14.6% vs. 12.2%, respectively) in a previous clinical trial [14]. PD-L1 positivity was confirmed in 60 (83.3%) of the 72 patients in our study based on the test results (Table 1), and their ORR was 11.7% (7/60) (data not provided). However, the role of PD-L1 expression has not been clarified, so it currently has limited value as a predictive biomarker [24]. 

HPV infection in cervical cancer is also considered to be a possible prognostic marker of ICI response, although the role of HPV infection has not yet been clarified. Cervical cancer is known to be an HPV-induced neoplasm, and several researchers have found that HPV positivity is positively correlated with increased PD-L1 expression in cervical cancer [10,25]. However, as previously described, because the role of PD-L1 expression as a prognostic marker is unclear, additional investigation is required to elucidate the specific contribution of HPV-induced cervical carcinogenesis.

In the present study, we examined the effect of several factors on ORR: HPV positivity, histology (squamous vs. non-squamous), number of prior lines of chemotherapy, ECOG status, PD-L1 positivity, and burden of tumor. Only an ECOG ≤1 showed a significant difference in the univariate analyses (OR, 14.651; 95% CI, 1.809–118.675; *p* = 0.012). The other factors showed no statistically significant differences (Table 4). In non-small cell lung cancer patients, poor PS (ECOG ≥2) has been suggested as a negative predictive factor for ICIs treatment [26] or not [27]. Studies on the treatment response according to PS have not been reported in cervical cancer patients, and prospective clinical trials are needed. MSI-H/MMRd data could be obtained for only 28 patients, so this factor was not analyzed in the present study.

### 4.4. Limitations

The limitations of this study stem mainly from its retrospective design and short follow-up time. The lack of an independent central radiologic and pathologic review could also be a confounding factor. Because the central pathologic review was not conducted, the MSI-H/MMRd and PD-L1 information of tumor was not obtained from all patients. Furthermore, the response assessment could not be centralized by an independent central radiologic review. We did not perform an evaluation based on immune RECIST or immune-related RECIST to assess the immune response. The AE evaluation was also conducted retrospectively based on chart review, which is probably why the frequency of AEs reported here was low compared to prospective clinical trials that evaluated AEs using strict criteria.

Nevertheless, to the best of our knowledge, this retrospective study of a relatively large, mainly Asian cohort is the first to evaluate the effectiveness and safety of pembrolizumab treatment in patients with recurrent cervical cancer in a real-world setting.

## 5. Conclusions

In summary, the present study showed that pembrolizumab treatment has modest antitumor activity in patients with recurrent or persistent cervical cancer, including effective activity especially in patients with favorable performance status, in real-world practice. Further studies are warranted to identify predictive biomarkers for immune checkpoint inhibitors in cervical cancer. 

## Figures and Tables

**Table 1:** Clinico-pathologic characteristics of the patients (*n* = 117).

Characteristic	No. (%)
Age, Years	
Median, range	53 (28–79)
ECOG performance status	
0	16 (13.7%)
1	37 (31.6%)
2	45 (38.5%)
3	16 (13.7%)
4	3 (2.6%)
FIGO stage at diagnosis	
I	31 (26.5%)
II	34 (29.1%)
III	22 (18.8%)
IV	27 (23.1%)
Unknown	3 (2.6%)
HPV test result	
Positive ^^^	57 (48.7%)
Negative	14 (12.0%)
Unknown	46 (39.3%)
PD-L1 expression ^*^	
1≤	60 (51.3%)
1>	12 (10.3%)
Unknown	45 (38.5%)
Histology	
Squamous cell carcinoma	88 (75.2%)
Adenocarcinoma	19 (16.2%)
Adenosquamous cell carcinoma	4 (3.4%)
Neuroendocrine cell carcinoma	4 (3.4%)
Glassy cell carcinoma	1 (0.9%)
Basaloid squamous cell carcinoma	1 (0.9%)
Target lesion size, mm ^#^	
Median, range	67 (10–529)
Previous radiation therapy	50 (42.7%)
(CC)RTx	38 (76.0%)
(CC)RTx + hysterectomy	12 (24.0%) ^@^
Previous surgery	62 (53.0%)
RH	17 (27.4%) ^%^
RH + (CC)RTx	45 (72.6%) ^&^
Number of previous lines of chemotherapy	
1	38 (32.5%)
2	43 (36.8%)
3	24 (20.5%)
4	8 (6.8%)
≥5	4 (3.4%)

ECOG, Eastern Cooperative Oncology Group; FIGO, International Federation of Gynecology and Obstetrics; HPV, human papillomavirus; N/A, non-available; PD-L1, programmed death-ligand 1; (CC)RTx, concurrent chemoradiation or radiation therapy; RH, radical hysterectomy. ^^^ presence of any type of high-risk human papillomavirus regardless of test type. ^*^ determined by either the tumor proportion score (TPS) or the combined positive score (CPS). ^#^ sum of the target lesions. ^@^ including 2 cases of pelvic exenteration. ^%^ including 2 cases of radical trachelectomy. ^&^ including 2 cases of pelvic exenteration followed by radiation.

**Table 2:** Tumor responses assessed by RECIST v.1.1 (*n* = 117).

Anti-Tumor Activity	Total Population	Favorable PS Group (ECOG ≤1)
	*n* = 117	*n* =53
Best overall response		
CR	3 (2.6%)	3 (5.7%)
PR	8 (6.8%)	7 (13.2%)
SD	28 (23.9%)	14 (26.4%)
PD	67 (57.3%)	26 (49.1%)
Not able to be assessed	11 (9.4%)	3 (5.7%)
Objective response rate	11 (9.4%)	10 (18.9%)
95% CI	4.8 to 16.2	9.4 to 32.0
Disease control rate	39 (33.3%)	24 (45.3%)
95% CI	24.9 to 42.6	31.6 to 59.6
Time to response, months ^#^		
Median (range)	2.8 (1.3–13.1)	3.0 (1.3–13.1)
Duration of response, months ^#,&^		
Median (range)	NR (8.9–NR)	8.9 (7.3–10.4)
Duration of response, months ^#,^*	(*n* = 11)	(*n* = 10)
≥6	6 (54.5%)	5 (50.0%)
≥9	4 (36.4%)	3 (30.0%)
≥12	2 (18.2%)	1 (10.0%)

CR, complete response; PR, partial response; SD, stable disease; PD, progressive disease; CI, confidence interval; NR, not reached; PS, performance status. ^#^ Evaluated in patients who had a response (*n* = 11 for total population, *n* = 10 for favorable PS group). ^&^ Estimated using Kaplan-Meier method. * Percentages as a fraction of the number of responders.

**Table 3:** Treatment-related adverse events in the total population (*n* = 117).

Adverse Event	Any Grade	Grade 3/4
Any AE	55 (47.0%)	8 (6.8%)
Hypothyroidism	9 (7.7%)	0
Fatigue	5 (4.3%)	0
Skin rash	5 (4.3%)	2 (1.7%)
Anemia	4 (3.4%)	1 (0.9%)
AST/ALT elevated	3 (2.6%)	1 (0.9%)
Nausea	3 (2.6%)	0
Abdominal pain	3 (2.6%)	0
Dyspnea	3 (2.6%)	0
Colitis	2 (1.7%)	1 (0.9%)
Neutropenia	2 (1.7%)	1 (0.9%)
Thrombocytopenia	2 (1.7%)	0
Hyperphosphatemia	2 (1.7%)	0
Hypoalbuminemia	2 (1.7%)	0
Renal insufficiency	2 (1.7%)	0
Cough	2 (1.7%)	0
Hyperthyroidism	1 (0.9%)	0
Pneumonitis with pulmonary edema	1 (0.9%)	1 (0.9%)
Constipation	1 (0.9%)	0
Vomiting	1 (0.9%)	0
Dizziness	1 (0.9%)	0
Guillain-Barré syndrome	1 (0.9%)	1 (0.9%)
Any AE leading to discontinuation		
Guillain-Barre syndrome	1	1
Colitis	1	1
Pneumonitis with pulmonary edema	1	1

AE, adverse events; AST, aspartate aminotransferase; ALT, alanine aminotransferase.

**Table 4:** Logistic regression analysis of predictive factors for the objective response rate.

Predictive Factors	Univariate Analysis	
	OR (95% CI)	*p*-Value
HPV test result		
Negative	1	
Positive	1.529 (0.169–13.842)	0.705
Histology		
Non-SqCC	1	
SqCC	3.590 (0.439–29.329)	0.233
Number of prior lines of chemotherapy		
≤2	1	
≥3	1.315 (0.328–5.263)	0.699
ECOG performance status		
≥2	1	
≤1	14.651 (1.809–118.675)	0.012
PD-L1 expression		
<20	1	
≥20	2.133 (0.440–10.338)	0.347
Burden of tumor ^#^		
<2cm	1	0.120
2cm≤ <5cm	1.429 (0.244–8.375)	
5cm≤ <10cm	0.270 (0.34–2.165)	
≥10cm	0.192 (0.016–2.363)	

OR, odds ratio; HPV, human papillomavirus; SqCC, squamous cell carcinoma; ECOG, Eastern Cooperative Oncology Group; PD-L1, programmed death-ligand 1. ^#^ sum of the target lesion.

**Table 5:** Therapeutic results of PD-1 inhibitors monotherapy in recurrent or advanced cervical cancer patients.

Study	Keynote028	Keynote158	Checkmate358	NRG-GY002	Present Study	
					Total population	Favorable PS group
Phase	IB	II	I/II	II	Retro	Retro
Drug	Pembrolizumab 10mg/kg q2w	Pembrolizumab 200mg q3w	Nivolumab 240mg q2w	Nivolumab 3mg/kg q2w	Pembrolizumab 200mg q3w	Pembrolizumab 200mg q3w
N	24	98	19	25	117	53
Age, median (age)	42	46	51	45	53	52
Histology of SqCC	23 (96%)	92 (94%)	19 (100%)	15 (60%)	88 (75%)	42 (79%)
ECOG ≤1	24 (100%)	98 (100%)	18 (95%)	25(100%)	53 (45%)	53 (100%)
Prior lines CTx ≥3	9 (38%)	30 (31%)	0	N/A	36(31%)	15 (28%)
Positive PD–L1 expression	24 (100%) ^*^	82 (84%) ^#^	10 (53%) ^%^	17(77%) ^#^	57(49%) ^$^	30 (57%) ^$^
BOR						
CR	0	3 (3%)	3 (16%)	0	3 (3%)	3 (6%)
PR	4 (17%)	9 (8%)	2 (10%)	1 (4%)	8 (7%)	7 (13%)
SD	3 (13%)	18 (18%)	8 (42%)	9 (36%)	28 (24%)	14 (26%)
PD	16 (67%)	55 (56%)	6 (32%)	11 (44%)	67 (57%)	26 (49%)
Not evaluable	1 (4%)	13 (13%)	0	4 (16%)	11 (0%)	3 (6%)
ORR	4 (17%)	12(12%)	5 (26%)	1 (4%)	11 (9%)	10 (19%)
DCR	7 (29%)	30 (31%)	13 (68%)	10 (40%)	39 (33%)	24 (45%)
Time to response, months Median (range)	1.9 (1.7–8.2)	2.1(1.6–4.1)	1.7 (1.6–1.9)	N/A	2.8 (1.4–13.4)	3.0 (1.4–13.4)
Duration of response, monthsMedian (range)	5.4 (4.1–7.5)	NR (≥3.7–≥18.6)	NR (23.3–29.5)	3.8	NR (8.9-NR)	8.9 (7.3–10.4)
AE ≥3	5 (21%)	12 (12%)	4(21%)	6 (24%)	8 (7%)	3 (6%)
PFS at 6m	21%	25%	36%	16%	30%	44%
OS at 6m, 12m	67%, 40%	75%, 41%	89%, 78%	78%	59%, 41%	84%, 57%

PS, performance status; Retro, retrospective study; SqCC, squamous cell carcinoma; ECOG, Eastern Cooperative Oncology Group; PD-L1, programmed death-ligand 1; CTx, chemotherapy; BOR, best overall response; CR, complete response; PR, partial response; SD, stable disease; PD, progressive disease; ORR, objective response rate; DCR, disease control rate; AE, adverse events; PFS, progression-free survival; OS, overall survival; N/A, non-available. ^*^ ≥1% of modified proportion score, ^#^ ≥1 of combined positive score (CPS), ^%^ ≥1% of tumor proportion score (TPS), ^$^ TPS ≥ 1% or CPS ≥ 1.

## Supplementary Materials

The following are available online at https://www.mdpi.com/2072-6694/12/11/3188/s1, Figure S1. Kaplan-Meier estimates of survival in the favorable performance status group (*n* = 53). (A) Progression-free survival, (B) Overall survival, Table S1. Participating institutions (*n* = 16), Table S2. Clinical summary of patients whose best overall response was complete or partial response (*n* = 11), Table S3. Tumor responses in patients with squamous cell carcinoma histology (*n* = 88).
