# Supplementary material for: Real-World Experience of Pembrolizumab Monotherapy in Patients with Recurrent or Persistent Cervical Cancer: A Korean Multi-Center Retrospective Study (KGOG1041)
Source: Cancers (Basel). 2020 Oct 29;12(11):3188. doi: 10.3390/cancers12113188 (PMC7693862; doi:10.3390/cancers12113188)
Supplement: Supplementary file 1 [file cancers-12-03188-s001.pdf]

cancers

MDPI

Article

Real-world experience of pembrolizumab monotherapy in patients with recurrent or persistent cervical cancer: A Korean multi-center retrospective study (KGOG1041)

Min Chul Choi, Yong-Man Kim, Jeong-Won Lee, Yong Jae Lee, Dong Hoon Suh, Sung Jong Lee, Taek Sang Lee, Maria Lee, Dong Choon Park, Min Kyu Kim, Jong-Min Lee, Seung-Hyuk Shim, Seob Jeon, Kyung Jin Min, Mi Kyung Kim, Bo Wook Kim, Jeong Yeol Park, Byoung-Gie Kim, Dae Yeon Kim, Moon-Hong Kim, Hyun-Soo Kim and Jung-Yun Lee

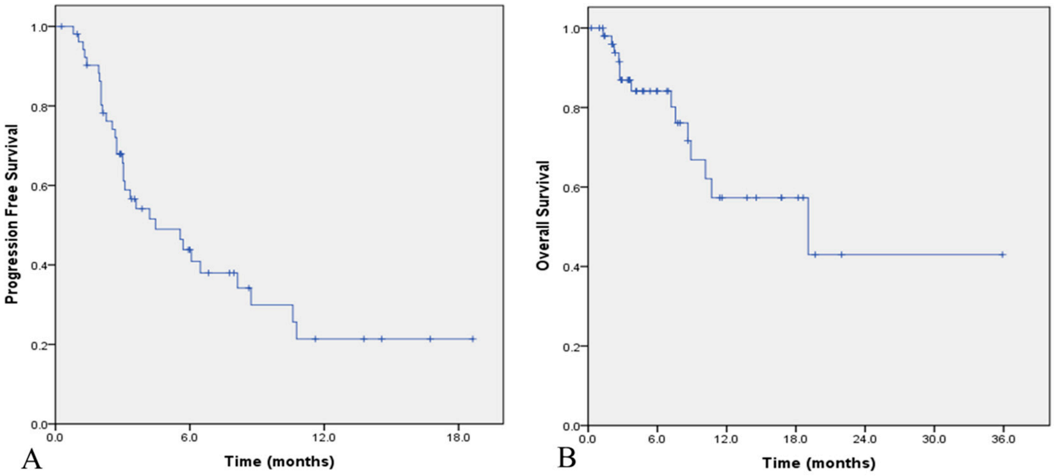

Figure S1. Kaplan-Meier estimates of survival in the favorable performance status group (n = 53). (**A**) Progression-free survival, (**B**) Overall survival.

Table S1. Participating institutions (n = 16).

Institution	Number of patients
Catholic Kwandong University International St. Mary's Hospital	2
Konkuk University Medical Center	2
Korea University Ansan Medical Center	2
Soonchunhyang University Hospital	2
Ewha Womans University Mokdong Hospital	1
Kyung Hee University Hospital at Gangdong	1
Samsung Changwon Hospital	3
St. Vincent's Hospital	4
Seoul National University Hospital	5
SMG-SNU Boramae Medical Center	5
Seoul St. Mary's Hospital	7
Seoul National University Bundang Hospital	7
Yonsei University Severance Hospital	9
CHA Bundang Medical Center	18
Asan Medical Center	19
Samsung Medical Center	30
Total	117

Table S2 Clinical summary of patients whose best overall response was complete or partial response (*n* = 11).

Case	Age	HPV	Histology	ECOG PS	No of lines of prior CTx	PD-L1 exp*	Cycles of pembrolizumab	BOR	Time to response(Mo)	DOR(Mo)	Current status
KDKH001	63	+	Squamous	1	2	N/A	#16 ongoing	CR	5.9	7.7	CR
SCH001	69	+	Squamous	0	1	N/A	#12 ongoing	CR	3.3	8.2	CR
BCHA008	72	N/A	Squamous	1	2	100%	#13	CR	1.9	14.6	CR
SNUBH003	43	N/A	Squamous	0	5	N/A	#19 ongoing	PR	13.1	1.2	AWD
SMC026	54	-	Squamous	1	1	30	#9 ongoing	PR	1.9	4.8	AWD
AMC017	49	N/A	Squamous	0	1	20	#8 ongoing	PR	5.8	2.8	AWD
AMC018	57	+	Squamous	0	3	N/A	#7 ongoing	PR	5.4	2.7	AWD
BCHA006	57	+	Squamous	3	2	90%	#24 ongoing	PR	1.5	19.6	CR[&]
BCHA013	46	+	Adenosq	0	4	17	#9 ongoing	PR	1.3	4.6	AWD
BRM001	42	N/A	Squamous	0	2	5%	#15	PR	2.8	7.7	AWD
BCHA010	52	+	Squamous	1	3	10%	#18	PR	1.9	1.7	Expired

HPV, human papillomavirus; ECOG PS, Eastern Cooperative Oncology Group performance status; CTx, chemotherapy; PD-L1 exp, expression of programmed death ligand 1; BOR, best overall response; Mo, months; DOR, duration of response; N/A, non-available; Squamous, squamous cell carcinoma; Adenosq, adenosquamous cell carcinoma; CR, complete response; PR, partial response; AWD, alive with disease. * expression of PD-L1 as determined by either tumor proportion score (TPS) or combined positive score (CPS) & after confirming a partial response of -90%, performed anterior pelvic exenteration after the 11th cycle of treatment to repair a vesico-uterine fistula. The patient stayed in complete response during the 24th cycle of treatment after the operation.

Table S3. Tumor responses in patients with squamous cell carcinoma histology (n = 88).

**Anti-tumor activity**	***n* = 88**
Best overall response	
CR	3 (3.4%)
PR	7 (8.0%)
SD	22 (25.0%)
PD	47 (53.4%)
Not able to be assessed	9 (10.2%)
Objective response rate	10 (11.4%)
95% CI	5.6 to 19.9
Disease control rate	32 (36.4%)
95% CI	26.4 to 47.3
Time to response, months	
Median (range)	3.0 (1.3–13.1)
Duration of response, months	
Median (range)	NR
Duration of response, months*	(n = 10)
≥6	6 (60%)
≥9	4 (40%)
≥12	2 (20%)

CR, complete response; PR, partial response; SD, stable disease; PD, progressive disease; CI, confidence interval; NR, not reached. * Percentages as a fraction of the number of responders.
